# One Health Approach in Serosurvey of *Toxoplasma gondii* in Former Black Slave (Quilombola) Communities in Southern Brazil and Among Their Dogs

**DOI:** 10.3390/tropicalmed8070377

**Published:** 2023-07-24

**Authors:** Giovanni Kalempa Panazzolo, Louise Bach Kmetiuk, Orlei José Domingues, João Henrique Farinhas, Fernando Rodrigo Doline, Danilo Alves de França, Nássarah Jabur Lot Rodrigues, Leandro Meneguelli Biondo, Rogério Giuffrida, Helio Langoni, Vamilton Alvares Santarém, Alexander Welker Biondo, Giovani Marino Fávero

**Affiliations:** 1 Graduate College of Pharmaceutical Sciences, State University of Ponta Grossa, Ponta Grossa 84030-900, PR, Brazilgmfavero@uepg.br (G.M.F.); 2 Graduate College of Cellular and Molecular Biology, Federal University of Paraná, Curitiba 81530-000, PR, Brazil; 3 Department of Veterinary Hygiene and Public Health, São Paulo State University, Botucatu 18618-681, SP, Brazil; danilo.franca@unesp.br (D.A.d.F.); nassarah.lot@unesp.br (N.J.L.R.);; 4 National Institute of the Atlantic Forest (INMA), Brazilian Ministry of Science, Technology and Innovation, Santa Teresa 29650-000, ES, Brazil; leandromet@gmail.com; 5 Laboratory of Veterinary Parasitology, Veterinary Teaching Hospital, University of Western São Paulo, Presidente Prudente 19001-970, SP, Brazil; rgiuffrida@unoeste.br (R.G.);

**Keywords:** brazilian *quilombos*, *quilombola*, one health, zoonosis

## Abstract

Brazilian *quilombos* are rural semi-isolated remnant communities of former black slaves and their descendants who traditionally maintained themselves through archaic subsistence livestock and agriculture practices and historically lacked specific public health policies. Although such individuals and their dogs may be exposed to zoonotic pathogens such as *Toxoplasma gondii*, no study to date has assessed these human-animal populations together. Populations in four different Brazilian *quilombos* in southern Brazil were evaluated. Overall, 93/208 people (44.7%) and 63/100 dogs (63.0%) were seropositive for IgG anti-*T. gondii* antibodies by indirect immunofluorescent antibody test (IFAT), 4/208 (1.9%) human samples seropositive for IgM anti-*T. gondii* antibodies, with a human-dog seropositivity ratio for IgG of 0.71. *Quilombola* individuals ingesting game meat were 2.43-fold more likely (95% CI: 1.05–5.9) to be seropositive. No risk factors were associated with seropositivity among dogs, thus suggesting that their exposure to *T. gondii* was random. Surprisingly, our research group had previously found an inverted human-dog ratio for *T. gondii* seropositivity of 2.54 in the urban area of a nearby major city. Because consumption of raw/undercooked game meat by *quilombola* individuals may have contributed to higher exposure, higher overall seroprevalence among dogs may have also indicated interaction with wildlife. Although these dogs may hunt wildlife without their owners’ awareness, the higher dog seropositivity may also be related to feeding from discarded food in the community or backyard livestock animals and drinking surface water contaminated with oocysts. Thus, wildlife cannot be singled out as the reason, and future studies should consider sampling water, soil, wildlife, and livestock tissues, to fully establish the source of infection in dogs herein.

## 1. Introduction

Toxoplasmosis has been described as a foodborne and waterborne disease caused by *Toxoplasma gondii* that is a matter of public and animal health concern worldwide [1]. *T. gondii* can infect all infected warm-blood species [2]. Most of these species have been considered intermediate hosts due to the asexual stage occurrence of *T. gondii* [2]. Domestic cats and other Felidae members are considered definitive hosts with *T. gondii* sexual stages and the capacity of oocyst shedding into feces in the environment and infecting intermediate hosts such as humans and dogs [2]. *T. gondii* development stages include the tachyzoite form (active and rapid division), bradyzoite (slow division and tissue cysts), and sporozoite (oocysts present in the environment) [3]. Ingestion of raw or undercooked meat with tissue cysts has been considered an important source of human *T. gondii* infection, including consumption of exotic and native species [1,4]. Additionally, consumption of water or vegetables containing oocysts, accidental ingestion of oocysts from contaminated soil, and vertical transmission have been recognized as other routes for human infection with *T. gondii* [5]. Seropositivity for *T. gondii* has been correlated with risk factors that include social vulnerability, lack of basic sanitary conditions, low income, and illiteracy [6,7]. In addition, individuals living in rural areas may be more exposed to *T. gondii* due to the vulnerable conditions, variety of domestic and wild intermediate hosts, unneutered cat population, and difficulty in accessing healthcare assistance [8,9].

Brazilian *quilombos* (*quilombola* as an adjective) have been defined as rural semi-isolated remnant communities that were formed by former black slaves originally during the time of slavery and persisted after abolition in 1888 [10]. Approximately 5972 such communities officially still exist nationwide, in which the inhabitants have preserved their African culture. *Quilombola* individuals have traditionally maintained themselves through subsistence agriculture [10]. The semi-isolation of these communities has been associated with their remote location, historical segregation implemented by European immigrant settlers, and the lack of specific public health policies [11,12]. Starting only in 2023, the Brazilian government created the Ministry of Racial Equality, which has become responsible for the planning, coordination, promotion, and execution of public policies toward racial equality and against racism [13].

Although *quilombola* individuals and their dogs may be exposed to *T. gondii*, no study to date has assessed these Brazilian *quilombola* populations. In addition, this scenario of overlapping risk factors for human, animal, and environmental health with regard to toxoplasmosis and associated risk factors demands the use of a One Health approach so that *T. gondii* infection can be effectively surveyed, analyzed, controlled, and prevented, as has already been established [14]. Accordingly, the aim of the present study was to assess the prevalence of IgG and IgM anti-*T. gondii* antibodies in humans, IgG anti-*T. gondii* antibodies in dogs, and associated risk factors in both for disease among inhabitants of four *quilombos* in southern Brazil.

## 2. Materials and Methods

### 2.1. Study Design and Area

The present study consisted of a cross-sectional survey of *T. gondii* exposure among *quilombola* individuals and their dogs. It was conducted in four different Brazilian *quilombos* located in rural areas of the state of Paraná, in southern Brazil, named Limitão, Mamans, Serra do Apon, and Tronco. These communities were established during the second half of the 19th century by individuals fleeing from slavery, which had mostly been associated with yerba mate plant cultivation and livestock activities. Similar to other Brazilian *quilombos*, the communities studied here were living through subsistence farming, backyard livestock-rearing, and handmade crafts. They were increasingly facing a struggle for their land, under pressure from forestry interests in surrounding commercial areas of *Eucalyptus* spp. wood production [15].

The studied population herein was estimated at 4000 unprivileged individuals with limited resources, officially recognized as lineal descendants of former black slavers and mostly distributed in unfertile, remote, and isolated areas of Paraná State [16]. The minimum sampling in such a scenario was calculated for 200 individuals, around 5% of the estimated *quilombola* population of Paraná State. Living in small and dispersed communities in rugged geographical areas of difficult access has historically granted safety and less recapture likelihood of fled individuals all at once during slavery. However, small communities around 50–60 km (31–37 miles) far from each other, connected by a few unmaintained gravel and sand roads at the time of the survey, have posted multiple logistical challenges. Human and dog samplings have required a total of six incursions, each one with previous door-to-door visits made by community leaders and city health agents. In addition, the four *quilombola* communities herein were assessed by a partnership with the Association of Rural *Quilombola* Communities at Castro County, founded in 2005 to strengthen member communities, preserve their African culture and fight for public policies [17]. Visits were conducted by a research task force of certified nurses, pharmaceuticals, veterinarians, and biologists, transported by a convoy of off-road vehicles carrying along tables, sampling chairs, field tents and sampling materials, and dog vaccines in monitored thermal boxes.

The study area at that time comprised both natural and degraded areas of the Atlantic Forest and Cerrado biomes, with a humid temperate climate of an average temperature of 17.5 °C and average rainfall of 1495 mm^3^.

This study was approved by the Ethics Committee for Human Health of the Brazilian Ministry of Health (protocol 53828121.1.0000.0105) and by the Ethics Committee for Animal Use (protocol 22.000075139-9) of the State University of Ponta Grossa.

### 2.2. Sample Size

A meta-analytic study with blood donors has estimated a 75% human toxoplasmosis prevalence in Brazil, one of the highest worldwide [18]. Thus, the minimum sampling size for logistic regression was calculated with a 50% estimated seroprevalence for the *quilombola* population, with a 0.05 probability of rejecting the null hypothesis (Type I error), 20% probability of failing to reject the null hypothesis under the alternative hypothesis (Type II error), and 1.5 minimum detectable odds ratio. Calculations based on available “Sample Size Calculators for designing clinical research” [19] resulted in a minimum of 194 individuals.

### 2.3. Sample Collection

A total of six field incursions were carried out between December 2021 and March 2022, and the investigation was performed in coordination with the Association of Rural *Quilombola* Communities in Castro County. All individuals voluntarily participated, signed a formal consent, and responded to an epidemiological questionnaire prior to samplings, applied by trained healthcare professionals. Illiterate persons were previously explained on contents and fingerprinted consent forms. For individuals under 18 years old, a parental signed consent form was required. A total of 10 mL of human blood samples were collected by cephalic puncture, performed by certified physicians and nurses. Dogs of *quilombola* communities herein had full outdoor access and exchanged multiple owners and households, making extremely difficult and unreliable any further statistical analysis of owner-dog risk for *T. gondii* infection. Thus, formal signed consent for dog sampling was obtained by one of these owners, and an epidemiological was questionnaire responded to by one or multiple owners. A total of 10 mL of blood samples were collected from dogs by jugular venipuncture, following physical restrain, and performed by certified veterinarians. All samples were collected in tubes without anticoagulant and centrifuged at 1500 revolutions per minute for five minutes. The serum was separated and stored at −20 °C until processing.

### 2.4. Serological Analysis

Detection of anti-*T. gondii* IgG and IgM antibodies were performed using the indirect immunofluorescent antibody test (IFAT) for human samples and anti-*T. gondii* IgG antibodies for dog samples, using two species-specific conjugates and positive controls as previously described [20].

Samples were tested for the detection of anti-*T. gondii* indirect fluorescent antibody test (IFAT). Anti-human specific IgG and IgM conjugate antibodies were used for human detection, while anti-dog IgG was used for dog detection. Immunofluorescence slides were sensibilized with the antigenic solution obtained from peritoneal washes in Swiss mice, previously inoculated (with 3 days), with 1 mL of enriched suspension of tachyzoites of *T. gondii* (strain RH). Before slide sensibilization, tachyzoites were inactivated with formaldehyde 0.1%.

Initially, human sera were submitted to detection of anti-human IgG, as previously established [21]. Each serum sample was diluted in phosphate-buffered saline solution (PBS) 0.01 M pH7.2 in the dilutions of 1:16, 1:64, 1:256, 1:1024, and 1:4096. In the same micro plaque, a similar procedure was performed for positive and negative control sera obtained in human patients at the routine service. Serum and correspondent dilutions were inserted into the previously sensibilized slides and incubated at 37 °C for 30 min. After washing in PBS 7.2, a commercial anti-human IgG antibody conjugated with fluorescein isothiocyanate (Bethyl Laboratories, Montgomery, TX, USA) was applied to the slides, serving as a secondary antibody reaction. This conjugate was diluted according to its previously established titer in Evans blue at 20 mg%. Slides were incubated again at 37 °C for 30 min. After slide drying, reading was performed at the immunofluorescence microscope with a 40X objective, considering as the final point of reaction the higher serum dilution in which complete and intense fluorescence was present in at least 50% of tachyzoites. Serum samples were then submitted to detection of anti-human IgM antibodies, following the same described procedure, but using commercially available specific anti-human IgM, also conjugated with fluorescein isothiocyanate (Bethyl Laboratories, Montgomery, TX, USA), as previously described [22]. Similar procedures were performed for IgM positive and negative control sera obtained in human patients at the routine service.

Dog serum samples were diluted in phosphate-buffered saline solution (PBS) 0.01 M pH7.2 in the dilutions of 1:16, 1:64, 1:256, 1:1024, and 1:4096, applied into previously sensibilized slides and incubated at 37 °C for 30 min. After washing in PBS 7.2, a commercial dog IgG antibody conjugated with fluorescein isothiocyanate (Zoonosis Control Center, Immunology Sector, São Paulo, SP, Brazil) was applied in the slides. Conjugate was diluted according to its titers, as previously established in Evans blue at 20 mg%. Slides were incubated again at 37 °C for 30 min. After slide drying, reading was performed at the immunofluorescence microscope with a 40X objective, considering as the final point of reaction the higher serum dilution in which complete and intense fluorescence was present in at least 50% of tachyzoites, as previously established [23]. A similar procedure was performed for IgM positive and negative control sera, obtained in dog patients at the routine service.

### 2.5. Epidemiological Data and Statistical Analysis

Human epidemiological analyses were performed based on a questionnaire about human exposure to *T. gondii*. This included questions on household location, sex, age, educational level, contact with soil, a water source used for consumption, human feces destination, dog or cat ownership, dog and cat ownership, consumption of raw or undercooked meat, consumption of raw or undercooked game meat, onychophagia, consumption of raw milk, travel outside the community, visual impairment, and history of miscarriage.

Dog epidemiological analyses were performed based on a questionnaire relating to dogs’ exposure to *T. gondii*. This included questions on household location, sex, age, mobility, type of food, consumption of raw or undercooked meat, hunting activities, and consumption of treated water.

The data collected were stratified into categorical outcomes and assessed for their association with *T. gondii* IgG seropositivity among humans and dogs, using Pearson’s chi-square test or Fisher’s exact test, if necessary. Variables that demonstrated a *p*-value < 0.20 in univariable models were selected for inclusion in a multivariable logistic regression analysis. The logistic model enabled the estimation of the odds ratio for each predictor variable and its corresponding 95% confidence interval. A significant level of 5% was applied to all statistical tests. The R Core Team software 4.1.0 (2022) was used to conduct all statistical analyses [24].

## 3. Results

**Figure 1:**
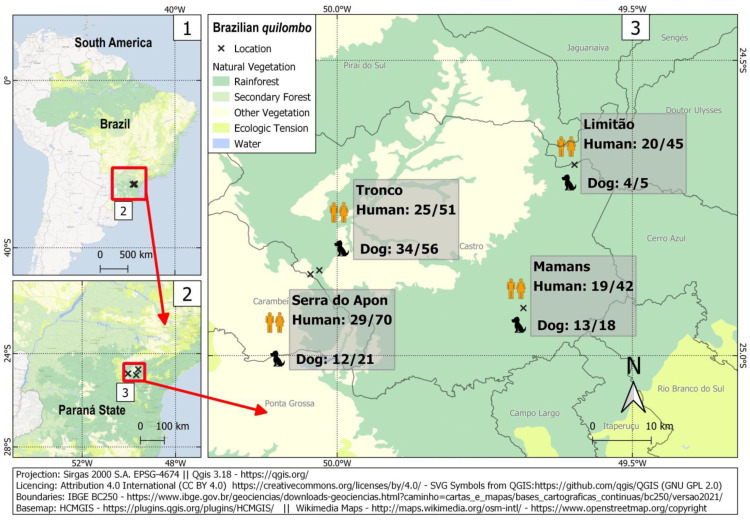
Sampling locations and frequency of occurrence of anti-*T. gondii* antibodies among *quilombola* individuals and their dogs in four Brazilian *quilombos* in southern Brazil.

A total of 208 *quilombola* individuals and 100 dogs were sampled for this study. In such a challenging situation, the sample size was considered reasonable, with adequate statistical power to assess associated risk factors to a 44% prevalence observed herein. Overall, 93/208 humans (44.7%; 95% CI: 38.1–51.5) and 63/100 dogs (63.0%; 95% CI: 53.2–71.8) were seropositive for IgG anti-*T. gondii* antibodies (Figure 1), with an overall human-dog seropositivity ratio of 0.71. Human seropositivity ranged from 41.4% (29/70; 95% CI: 30.6–53.1) in the Serra do Apon community to 49.0% (25/51; 95% CI: 35.9–62.3) in the Tronco community. Among pregnant women, 2/5 (40.0%) were seropositive. All samples were tested for IgM antibodies, with 4/208 (1.9%) seropositive samples from three different communities. These IgM-positive individuals comprised one seven-year-old boy and three adults (one man and two non-pregnant women). The presence of both IgG and IgM was found in one out of these four samples.

Several of the potential risk factors for *T. gondii* exposure among *quilombola* individuals that were tested for association were found not to be statistically significant, as follows: household location (*p* = 0.875), sex (*p* = 0.801), age (*p* = 0.166), educational level (*p* = 0.347), contact with soil (*p* = 0.333), water source used for consumption (*p* = 0.408), human feces destination (*p* = 0.645), dog or cat ownership (0.181), consumption of raw or undercooked meat (*p* = 0.882), consumption of raw or undercooked game meat (*p* = 0.068), onychophagia (*p* = 1), consumption of raw milk (*p* = 0.834), travel outside the community (*p* = 0.281), visual impairment (*p* = 0.436) and history of miscarriage (*p* = 1). From the logistic regression, individuals who declared that they consumed raw or undercooked game meat were 2.43-fold more likely (95% CI: 1.05–5.9) to be seropositive for *T. gondii*. Although age (*p* = 0.166) and dog or cat ownership (*p* = 0.181) were considered to be appropriate for multivariate analysis, the variables were not found to be significant according to the final logistic model (Table 1). Additionally, *quilombola* individuals have reported hunting white-lipped peccaries (*Pecari tajacu*), coatis (*Nasua nasua*), armadillos (*Dasypus* sp.), opossums (*Didelphis aurita*), capybaras (*Hydrochoerus hydrochaeris*), and wild boars (*Sus scrofa*).

Except for the household location, sex, and age variables, an evaluation of associated risk was obtained with a sample size of less than 208 due to the loss of information given by individuals (Table 1).

Dog seropositivity ranged from 57.1% in Serra do Apon (12/21; 95% CI: 36.5–75.5) to 80.0% (4/5; 95% CI: 37.6–96.4) in Limitão.

The following potentially associated risk factors for *T. gondii* exposure among dogs were found not to be statistically significant: household location (*p* = 0.634), sex (*p* = 1.0), age (*p* = 0.604), mobility (*p* = 0.950), type of food (*p* = 0.452), consumption of raw meat (*p* = 0.981), hunting activities (*p* = 0.649) and consumption of treated water (*p* = 1.0). None of these variables were considered appropriate for logistic regression analysis (*p* > 0.2) (Table 2). Loss of information given by individuals limited calculation of associated risk factors when o a sample lower than 100, except for household location, sex, and type of food variables.

## 4. Discussion

To the authors’ knowledge, this was the first study assessing *T. gondii* exposure among *quilombola* individuals and their dogs. These individuals have historically lived under conditions of high socioeconomic vulnerability, such as low family income and education levels, difficulty in accessing potable drinking water and healthcare services [25,26,27], and high levels of food insecurity [28].

The seropositivity rate among the human individuals surveyed here (44.7%) was higher than the pooled estimated seroprevalence of 36% for the general population worldwide, as determined through a global meta-analysis [29]. Higher *T. gondii* seroprevalence was also observed in another vulnerable population in a nearby city (526/715; 73.57%), where it was associated with high social vulnerability indicators such as illiteracy, unemployment and lack of basic sanitary conditions [6]. In addition, higher seropositivity was found among female individuals (102/194; 52.6%) in the population of a rural settlement in the state of São Paulo with a monthly income lower than US$ 300 [30]. Contrasting with the 0.71 human-dog seroprevalence ratio in the present study, an inverted 2.54 ratio was found in the urban area of Londrina, a half-million people city far 270 km (168 miles) from *the quilombola* communities herein [31]. In this study, 248/597 (41.54%) owners and 119/729 (16.32%) dogs were seropositive by IFAT to anti-*T. gondii*, indicating a 3.58-fold higher exposure of dogs to infection in *quilombola* communities when compared to urban areas of a major Brazilian city. While dog owners have shown similar seroprevalence, differences in *T. gondii* seroprevalence between dogs in urban settings (16.32%) and dogs in *quilombola* communities (63.0%) have indicated much higher exposure to associated risk factors in *quilombola* dogs. The higher dog seropositivity herein may be related to feeding on discarded food by the community or backyard livestock animals and drinking surface water contaminated with oocysts. Thus, even recognizing that hunting and wild wandering on their own may have led to such infection and misreporting in the epidemiological questionnaire, wildlife should not be singled out as the reason for high dog serology.

Toxoplasmosis has a complex epidemiology. It is transmitted through ingestion of oocysts that were shed in the feces of definitive feline hosts and subsequently contaminated water, soil, and crops or through consumption of cysts in raw or undercooked meat from intermediate hosts [32], particularly game meat [33]. Logistic multiple regression revealed that the habit of ingesting game meat was the sole risk factor for seropositivity among *quilombola* human residents. Although such findings may be extrapolated on dog exposure through dog hunting and wandering activities on wildlife, undertaken without owner presence or knowledge, dogs could, in fact, be eating discarded food within the community, including meat, which may not be from wildlife, and/or drinking from surface water sources, contaminated with oocysts. As mentioned, dogs in the indigenous communities herein were mostly free-range, similar to the community or stray dogs, with some connection but little care from individual owners in terms of feeding and providing water and full access throughout the community, including water sources, garbage, and sewage.

Although such other causes should be considered, hunting activities have been reported to be common among *quilombola* individuals, and such activities form a source of animal protein for remote communities in Brazil [34]. In such a scenario, the present study has corroborated with a study on hunting and ecology of mammals among *quilombola* individuals in southeastern Brazil revealed that the animals hunted were mostly small mammals such as white-lipped peccaries, coatis, armadillos, and opossums [35]. The individuals surveyed here reported that they hunted small, medium, and large wild animals, including native capybaras and exotic invasive species such as wild boars. In studies on *T. gondii* seropositivity among capybaras, although 16/26 healthy free-range capybaras (61.5%) caught in a large nearby city, 160 km (100 miles) from the present study, were seropositive [36], another study within the same state of Paraná found seropositivity in only 4/21 free-range capybaras (16.1%) and 1/10 captive capybaras in a zoo (10.0%) [37].

Although also a source of *T. gondii* infection [38], free-range wild boars in the same area were less exposed to *T. gondii* than hunters and their hunting dogs [39]. On the other hand, captive wild boars maintained in backyard farms were more likely seropositive, probably due to the presence of domestic cats in such more anthropized areas [39].

While a study in the state of Paraná showed seropositivity for *T. gondii* in 7/22 collared peccaries (31.8%) and in 0/6 white-lipped peccaries (0%) that were kept in captivity [37], a study conducted in Peru found that 90/101 white-lipped peccaries (89.1%) that had contact with domestic animals were seropositive [40]. Furthermore, infective *T. gondii* was isolated from black-eared opossums living in an urban area in southeastern Brazil [41]. Although occurrences of contaminated meat have mostly been irregularly distributed, with low levels of tissue cysts, such that individuals eating infected meat may escape infection [42], the frequent hunting activities of *quilombola* individuals and their consumption of raw/undercooked game meat may have contributed to higher exposure to *T. gondii*. Again, further surveys should take into account other sources, such as garbage, sewers, water, and soil, to pinpoint the role of each potential source in dog infection.

Living in rural areas, as observed here, has been correlated with *T. gondii* exposure in Brazil. In another study, seropositivity (183/344; 53.2%) was associated with the consumption of undercooked meat in such areas [8]. Raw or undercooked meat intake was considered to be such a strong *T. gondii* risk factor that it could increase the risk 1.2–1.3-fold and the odds of infection 1.7–3.0-fold, regardless of which animal species was consumed [8]. Likewise, given that Brazilian *quilombola* individuals live through subsistence livestock and agriculture activities, their backyard pigs and poultry may be more exposed to *T. gondii*, usually associated with the presence of cats and unreliable water sources [43]. Lastly, no adequate thermal methods (freezing or heating) for the inactivation of tissue cysts were used in game meat for most sampled humans. The rudimentary animal slaughter methods used predispose *quilombola* communities to the persistence and transmission of *T. gondii* [4].

All the remote communities surveyed here reported that untreated water sources were used for consumption, with no statistical difference in their use between the river, well, or spring sources. As previously shown in a review, the high frequency of *T. gondii* outbreaks in Brazil was correlated with environmental contamination, particularly with regard to water and poor hygiene, and socioeconomic conditions [42]. Moreover, other outbreaks worldwide have been reported in small and remote communities that share only one source of water [42]. Thus, in addition to the high seropositivity, predisposing conditions in the remote communities surveyed here may have facilitated the occurrence of *T. gondii* outbreaks.

The prevalence of IgM found here (4/208; 1.9%) was lower than the estimated seroprevalence (4.0%) revealed by a recent meta-analysis [44]. Anti-*T. gondii* IgM antibodies have been widely used for early detection of acute toxoplasmosis, particularly among high-risk population groups, including pregnant women (due to congenital infection) and immunodeficient individuals (due to reactivation of latent infection and neurological impairment) [29]. In Brazil, positivity for IgM antibodies was detected in 21/194 individuals (10.8%) in a rural settlement in the southeastern region, with no associated risk factors to explain these findings [30]. Although IgM forms part of the initial immune response, results regarding the presence of IgM need to be carefully interpreted due to the short lifespan of this antibody (less than 14 days) and the decreasing production of this antibody over time [45]. Only one person in the present study showed concomitant positivity for both IgM and IgG antibodies, which thus suggests that there was a very low occurrence of acute *T. gondii* infection. Fortunately, all the pregnant women were seronegative for IgM antibodies, despite high IgG seropositivity (23/43; 53.8%) among women within their reproductive age range (we considered this to range from 18 to 40 years of age). This high IgG seropositivity may indicate pregnancy risk and a need for serological follow-up during the gestational period in order to avoid congenital transmission.

The seropositivity rate among the dogs of the present study (63.0%) was similar to what has been found in previous Brazilian serosurveys, as observed in 150/247 dogs (54.7%) on the island of Fernando de Noronha [46]; 48/107 dogs (43.1%) living in riverside communities in central-western Brazil [47]; 8/26 neighborhood dogs (30.7%) [48], 49/157 hunting dogs (31.2%) [39] and 66/283 dogs (23.3%) living on islands and mainland coastal areas [49]. In this last study, conducted by our research group, the presence of anti-*T. gondii* antibodies in dogs was associated with seropositivity among their owners (*p* = 0.008; OR = 2.81) due to sharing of food and water intake between owners and their dogs, as reported by the owners. A dog serosurvey conducted during one of the largest toxoplasmosis outbreaks to date worldwide (which occurred in southern Brazil) revealed that 185/1159 dogs (16.0%) were seropositive before the outbreak, while 466/1086 (42.9%) were seropositive after the outbreak. This emphasized the likely similar exposure of dogs to contaminated water sources [50]. Dogs herein had multiple exchange owners of relative families and circulated among nearby households. Thus, as dog samples herein were not matched to tested owners or households, analysis of owner-dog *T. gondii* seropositivity was not performed.

Although no associated risk factor for *T. gondii* infection was observed among the dogs surveyed here, including sex, age, ownership, nutrition, ingestion of raw or undercooked meat, hunting habit and source of water, *quilombola* dogs living in contact with other domestic animals and wildlife may be more exposed to diseases, particularly through infection from vector-borne pathogens [51]. Considering that 29/87 (33.3%) of the dogs surveyed (87/100 obtained information regarding dogs) here were used during hunting activities, this scenario may also favor *T. gondii* infection due to greater access to trails, forests, and rivers and contact with soil. Not surprisingly, hunting activity was considered to be a risk factor associated with *T. gondii* infection among dogs in Spain [52], and hunters in Brazil have reported that they offered raw meat from wild boars to their hunting dogs after slaughtering the animals [39]. Thus, *quilombola* dogs may also hunt small prey without their owners’ awareness, and ingestion of these birds and small rodents may represent a potential source for *T. gondii* infection.

Although satisfying the minimum calculated sample size, limitations in the present study may include the relatively low sampling, mostly due to limitations on individual house-to-house assessment, free time of volunteers for sampling and questionnaire response, and willingness to participate, which may have generated insufficient data to provide the basis for a representative statistical description and analyses. In addition, data obtained by the epidemiological questionnaires were based on personal perception at the time of the survey, which may not reflect the true information, particularly from children, elderly, and illiterate individuals about themselves and their dogs.

Lastly, the human-dog seropositivity ratio resulting from this One Health approach may be used to compare human and dog exposure and associated risk factors for *T. gondii* infection, as well as changes to the ratio according to different environmental settings. Further studies should also include environmental surveys of *T. gondii*, including molecular detection in samples of water, soil, and food (particularly livestock and game meat).

## 5. Conclusions

In summary, high seroprevalence was observed in both the human and the dog populations in these Brazilian quilombos. Furthermore, the results from this study suggest that consumption of game meat may play a pivotal role in the transmission of toxoplasmosis in the human *quilombola* population, but dog exposure may include a series of potential causes and should be further investigated. The information presented here should be provided in the form of public health education in order to mitigate the risk of toxoplasmosis among *quilombola* individuals and their dogs.

## Figures and Tables

**Table 1:** Associated risk factors for anti-*Toxoplasma gondii* (IgG) antibodies among *quilombola* individuals (n = 208) living in Brazilian *quilombos* in southern Brazil.

			Univariate Analysis		Multivariate Analysis	
Variables	Seropositive n (%)	Seronegative n (%)	OR (95% CI)	*p*-Value	OR (95% CI)	*p*-Value
Household location	n = 93	n = 115		0.875		
Limitão	20 (21.5)	25 (21.7)	1.0			
Mamans	19 (20.4)	23 (20.0)	1.03 (0.44–2.43)			
Serra do Apon	29 (31.2)	41 (35.7)	0.88 (0.41–1.90)			
Tronco	25 (26.9)	26 (22.6)	1.20 (0.53–2.71)			
Sex	n = 93	n = 115		0.801		
Female	55 (59.1)	71 (61.7)	1.0			
Male	38 (40.9)	44 (38.3)	1.12 (0.64–1.97)			
Age (years)	n = 93	n = 115		0.166		
<6	4 (4.3)	7 (6.1)	1.0		1.0	
6 to 17	17 (18.3)	18 (15.7)	1.62 (0.40–7.42)		2.06 (0.45–11.3)	0.336
18 to 59	65 (69.9)	70 (60.9)	1.60 (0.45–6.58)		1.61 (0.4–8.0)	0.519
>59	7 (7.5)	20 (17.4)	0.62 (0.13–3.08)		0.61 (0.12–3.58)	0.557
Educational level *	n = 91	n = 113		0.347		
Illiterate	13 (14.3)	20 (17.7)	1.0			
Elementary	72 (79.1)	90 (79.6)	1.23 [0.57–2.70]			
High school	6 (6.6)	3 (2.7)	2.93 (0.63–17.0)			
Contact with soil *	n = 90	n = 112		0.333		
No	4 (4.4)	10 (8.9)	1.0			
Yes	86 (95.6)	102 (91.1)	2.06 (0.65–7.97)			
Water source used for consumption *	n = 81	n = 103		0.408		
River	13 (16.0)	10 (9.7)	1.0			
Well	23 (28.4)	34 (33.0)	0.53 (0.19–1.41)			
Spring	45 (55.6)	59 (57.3)	0.59 (0.23–1.48)			
Human feces destination *	n = 92	n = 112		0.645		
Dry latrine	6 (6.5)	4 (3.6)	1.0			
Environment	37 (40.2)	45 (40.2)	0.56 (0.13–2.16)			
Septic tank	49 (53.3)	63 (56.2)	0.53 (0.12–2.01)			
Dog or cat ownership *	n = 88	n = 112		0.186		
No	6 (6.8)	3 (2.7)	1.0		1.0	
Yes	82 (93.2)	109 (97.3)	0.39 (0.08–1.56)		0.27 (0.05–1.15)	0.090
Consumption of raw or undercooked meat *	n = 92	n = 112		0.882		
No	84 (91.3)	104 (92.9)	1.0			
Yes	8 (8.7)	8 (7.1)	1.24 (0.43–3.55)			
Consumption of raw or undercooked game meat *	n = 91	n = 112		0.068		0.042
No	74 (81.3)	102 (91.1)	1.0		1.0	
Yes	17 (18.7)	10 (8.9)	2.32 (1.01–5.58)		2.43 (1.05–5.9)	
Onychophagia *	n = 88	n = 110		1		
No	65 (73.9)	82 (74.5)	1.0			
Yes	23 (26.1)	28 (25.5)	1.04 (0.54–1.97)			
Consumption of raw milk	n = 89	n = 112		0.834		
No	21 (23.6)	29 (25.9)	1.0			
Yes	68 (76.4)	83 (74.1)	1.13 (0.59–2.18)			
Travel outside the community *	n = 84	n = 114		0.281		
No	76 (90.5)	96 (84.2)	1.0			
Yes	8 (9.5)	18 (15.8)	1.78 (0.73–4.32)			
Visual impairment *	n = 90	n = 110		0.436		
No	57 (63.3)	62 56.9)	1.0			
Yes	33 (36.7)	47 (43.1)	0.77 (0.43–1.36)			
History of miscarriage *	n = 39	n = 52		1		
No	31 79.5)	42 (80.8)	1.0			
Yes	8 (20.5)	10 (19.2)	0.90 (0.32–2.55)			

* Missing information.

**Table 2:** Associated risk factors for anti-*Toxoplasma gondii* (IgG) antibodies among dogs (N = 100) living in Brazilian *quilombos*.

	Seropositive n (%)	Seronegative n (%)	Univariate Analysis	
Variables	63 (63.0)	37 (37.0)	OR (95% CI)	*p*-Value
Household location	n = 63	n = 37		0.634
Limitão	4 (6.4)	1 (2.7)	1.0	
Mamans	13 (20.6)	5 (13.5)	0.71 (0.02–6.99)	
Serra do Apon	12 (19.0)	9 (24.3)	0.37 (0.01–3.32)	
Tronco	34 (54.0)	22 (59.5)	2.33 (030–66.8)	
Sex	n = 63	n = 37		1.0
Female	22 (34.9)	13 (35.1)	1.0	
Male	41 (65.1)	24 (64.9)	1.01 (0.42–2.37)	
Age (years) *	n = 63	n = 36		0.604
<1	16 (25.4)	7 (19.4)	1.0	
1 to 8	43 (68.3)	28 (77.8)	0.68 (0.23–1.83)	
>8	4 (6.4)	1 (2.8)	1.59 (0.18–49.1)	
Mobility *	n = 52	n = 32		0.950
Not limited to peridomestic area	13 (25.0)	7 (21.9)	1.0	
Limited to peridomestic area	39 (75.0)	25 (78.1)	0.85 (0.28–2.40)	
Type of food	n = 63	n = 37		0.452
Homemade food	16 (25.4)	14 (37.8)	1.0	
Dry food	42 (66.7)	21 (56.8)	1.74 (0.71–4.29)	
Both	5 (7.9)	2 (5.4)	2.07 (0.36–18.1)	
Consumption of raw or undercooked meat *	n = 45	n = 26		0.981
No	21 (46.7)	13 (50.0)	1.0	
Yes	24 (53.3)	13 (50.0)	1.14 (0.43–3.05)	
Hunting activities *	n = 55	n = 32		0.649
No	40 (72.7)	21 (65.6)	1.0	
Yes	15 (27.3)	11 (34.4)	0.72 (0.28–1.88)	
Consumption of treated water *	n = 61	n = 33		1.0
No	35 (57.4)	19 (57.6)	1.0	
Yes	26 (42.6)	14 (42.4)	1.01 (0.42–2.41)	

* Missing information.

## Data Availability

Not applicable.

## References

[1] Jones J.L., Dubey J.P. (2012). Foodborne Toxoplasmosis. Clin. Infect. Dis. Off. Publ. Infect. Dis. Soc. Am..

[2] Attias M., Teixeira D.E., Benchimol M., Vommaro R.C., Crepaldi P.H., De Souza W. (2020). The Life-Cycle of Toxoplasma Gondii Reviewed Using Animations. Parasit. Vectors.

[3] Al-Malki E.S. (2021). Toxoplasmosis: Stages of the Protozoan Life Cycle and Risk Assessment in Humans and Animals for an Enhanced Awareness and an Improved Socio-Economic Status. Saudi J. Biol. Sci..

[4] Marín-García P.-J., Planas N., Llobat L. (2022). Toxoplasma Gondii in Foods: Prevalence, Control, and Safety. Foods.

[5] CDC—Centers for Disease Control and Prevention (2019). Toxoplasmosis—Epidemiology & Risk Factors.

[6] Mareze M., do Nascimento Benitez A., Brandão A.P.D., Pinto-Ferreira F., Miura A.C., Martins F.D.C., Caldart E.T., Biondo A.W., Freire R.L., Mitsuka-Breganó R. (2019). Socioeconomic Vulnerability Associated to Toxoplasma Gondii Exposure in Southern Brazil. PLoS ONE.

[7] Francisco F.D.M., De Souza S.L.P., Gennari S.M., Pinheiro S.R., Muradian V., Soares R.M. (2006). Seroprevalence of Toxoplasmosis in a Low-Income Community in the São Paulo Municipality, SP, Brazil. Rev. Inst. Med. Trop. Sao Paulo.

[8] Araújo A.C., Villela M.M., Sena-Lopes Â., da Rosa Farias N.A., de Faria L.M.J., da Costa Avila L.F., Berne M.E.A., Borsuk S. (2018). Seroprevalence of Toxoplasma Gondii and Toxocara Canis in a Human Rural Population of Southern Rio Grande Do Sul. Rev. Inst. Med. Trop. Sao Paulo.

[9] Mahdy M.A.K., Alareqi L.M.Q., Abdul-Ghani R., Al-Eryani S.M.A., Al-Mikhlafy A.A., Al-Mekhlafi A.M., Alkarshy F., Mahmud R. (2017). A Community-Based Survey of Toxoplasma Gondii Infection among Pregnant Women in Rural Areas of Taiz Governorate, Yemen: The Risk of Waterborne Transmission. Infect. Dis. Poverty.

[10] Maria da Silva G., Souza B.O. (2022). Quilombos in Brazil and the Americas: Black Resistance in Historical Perspective. Agrar. South J. Polit. Econ..

[11] da Silva Gomes W., Gurgel I.G.D., Fernandes S.L. (2022). Determinação Social Da Saúde Numa Comunidade Quilombola: Análise Com a Matriz de Processos Críticos. Serviço Soc. Soc..

[12] Freitas D.A., Caballero A.D., Marques A.S., Hernández C.I.V., Antunes S.L.N.O. (2011). Saúde e Comunidades Quilombolas: Uma Revisão Da Literatura. Rev. CEFAC.

[13] Ministério Da Igualdade Racial. https://www.gov.br/igualdaderacial/pt-br.

[14] Pettan-Brewer C., Martins A.F., de Abreu D.P.B., Brandão A.P.D., Barbosa D.S., Figueroa D.P., Cediel N., Kahn L.H., Brandespim D.F., Velásquez J.C.C. (2021). From the Approach to the Concept: One Health in Latin America-Experiences and Perspectives in Brazil, Chile, and Colombia. Front. Public Health.

[15] Campos M.D.C., Gallinari T.S. (2017). Permanência e Resistência Das Comunidades Remanescentes de Quilombos No Paraná. Geosaberes.

[16] Comunidades Quilombolas No Paraná A Escravidão No Brasil.

[17] COMUNIDADES NEGRAS RURAIS DE CASTRO | Ancestralidade Africana. https://ancestralidadeafricana.org.br/quilombos/comunidade-quilombola-serra-do-apon/.

[18] Foroutan-Rad M., Majidiani H., Dalvand S., Daryani A., Kooti W., Saki J., Hedayati-Rad F., Ahmadpour E. (2016). Toxoplasmosis in Blood Donors: A Systematic Review and Meta-Analysis. Transfus. Med. Rev..

[19] Logistic Regression—Sample Size | Sample Size Calculators. https://sample-size.net/logistic-regression-sample-size/.

[20] Camargo M.E. (1973). Introdução Às Técnicas de Imunofluorescência.

[21] Villard O., Cimon B., L’Ollivier C., Fricker-Hidalgo H., Godineau N., Houze S., Paris L., Pelloux H., Villena I., Candolfi E. (2016). Help in the Choice of Automated or Semiautomated Immunoassays for Serological Diagnosis of Toxoplasmosis: Evaluation of Nine Immunoassays by the French National Reference Center for Toxoplasmosis. J. Clin. Microbiol..

[22] Murat J.-B., Hidalgo H.F., Brenier-Pinchart M.-P., Pelloux H. (2013). Human Toxoplasmosis: Which Biological Diagnostic Tests Are Best Suited to Which Clinical Situations?. Expert Rev. Anti. Infect. Ther..

[23] Jittapalapong S., Nimsupan B., Pinyopanuwat N., Chimnoi W., Kabeya H., Maruyama S. (2007). Seroprevalence of Toxoplasma Gondii Antibodies in Stray Cats and Dogs in the Bangkok Metropolitan Area, Thailand. Vet. Parasitol..

[24] R: The R Project for Statistical Computing. https://www.r-project.org/.

[25] Hélia de Lima Sardinha A., Bruna Arruda Aragão F., Morais Silva C., Márita Ribeiro Rodrigues Z., Dias Reis A., van Deursen Varga I., Francisca Bruna Arruda Aragão C. (2019). Quality of Life of Elderly Quilombolas in the Brazilian Northeast. Rev. Bras. Geriatr. e Gerontol..

[26] Quaresma F.R.P., da Silva Maciel E., Barasuol A.M., Pontes-Silva A., Fonseca F.L.A., Adami F. (2022). Quality of Primary Health Care for Quilombolas’ Afro-Descendant in Brazil: A Cross-Sectional Study. Rev. Assoc. Med. Bras..

[27] Santos E.N.A., Magalhães P.K.A., Santos A.M., Correia M.S., Santos J.C.S., Carvalho Neto A.P.M., Souza M.A., Lima R.F., Fonseca S.A., Ferreira-Júnior G.C. (2022). Quality of Life of Women from a Quilombola Community in Northeastern Brazil. Braz. J. Biol..

[28] Gubert M.B., Segall-Corrêa A.M., Spaniol A.M., Pedroso J., Coelho S.E.D.A.C., Pérez-Escamilla R. (2017). Household Food Insecurity in Black-Slaves Descendant Communities in Brazil: Has the Legacy of Slavery Truly Ended?. Public Health Nutr..

[29] Rahmanian V., Rahmanian K., Jahromi A.S., Bokaie S. (2020). Seroprevalence of Toxoplasma Gondii Infection: An Umbrella Review of Updated Systematic Reviews and Meta-Analyses. J. Fam. Med. Prim. care.

[30] Prestes-Carneiro L.E., Rubinsky-Elefant G., Ferreira A.W., Araujo P.R., Troiani C., Zago S.C., Kaiahara M., Sasso L., Iha A., Vaz A. (2013). Seroprevalence of Toxoplasmosis, Toxocariasis and Cysticercosis in a Rural Settlement, São Paulo State, Brazil. Pathog. Glob. Health.

[31] do Nascimento Benitez A., Martins F.D.C., Mareze M., Santos N.J.R., Ferreira F.P., Martins C.M., Garcia J.L., Mitsuka-Breganó R., Freire R.L., Biondo A.W. (2017). Spatial and Simultaneous Representative Seroprevalence of Anti-Toxoplasma Gondii Antibodies in Owners and Their Domiciled Dogs in a Major City of Southern Brazil. PLoS ONE.

[32] Smith N.C., Goulart C., Hayward J.A., Kupz A., Miller C.M., van Dooren G.G. (2021). Control of Human Toxoplasmosis. Int. J. Parasitol..

[33] Castro-Scholten S., Cano-Terriza D., Jiménez-Ruiz S., Almería S., Risalde M.A., Vicente J., Acevedo P., Arnal M.C., Balseiro A., Gómez-Guillamón F. (2021). Seroepidemiology of Toxoplasma Gondii in Wild Ruminants in Spain. Zoonoses Public Health.

[34] Corrêa N., Silva H. (2021). Da Amazônia Ao Guia: Os Dilemas Entre a Alimentação Quilombola e as Recomendações Do Guia Alimentar Para a População Brasileira. Saúde e Soc..

[35] Prado H.M., Da Silva R.C., Schlindwein M.N., Murrieta R.S.S. (2020). Ethnography, Ethnobiology and Natural History: Narratives on Hunting and Ecology of Mammals among Quilombolas from Southeast Brazil. J. Ethnobiol. Ethnomed..

[36] Truppel J.H., Reifur L., Montiani-Ferreira F., Lange R.R., de Castro Vilani R.G.D., Gennari S.M., Thomaz-Soccol V. (2010). Toxoplasma Gondii in Capybara (Hydrochaeris Hydrochaeris) Antibodies and DNA Detected by IFAT and PCR. Parasitol. Res..

[37] Ullmann L.S., Gravinatti M.L., Yamatogi R.S., dos Santos L.C., de Moraes W., Cubas Z.S., Camossi L.G., de Barros Filho I.R., Langoni H., da Costa Vieira R.F. (2017). Serosurvey of Anti-Leptospira sp. and Anti-Toxoplasma Gondii Antibodies in Capybaras and Collared and White-Lipped Peccaries. Rev. Soc. Bras. Med. Trop..

[38] Rostami A., Riahi S.M., Fakhri Y., Saber V., Hanifehpour H., Valizadeh S., Gholizadeh M., Pouya R.H., Gamble H.R. (2017). The Global Seroprevalence of Toxoplasma Gondii among Wild Boars: A Systematic Review and Meta-Analysis. Vet. Parasitol..

[39] Machado F.P., Kmetiuk L.B., Teider-Junior P.I., Pellizzaro M., Yamakawa A.C., Martins C.M., van Wilpe Bach R., Morikawa V.M., de Barros-Filho I.R., Langoni H. (2019). Seroprevalence of Anti-Toxoplasma Gondii Antibodies in Wild Boars (Sus Scrofa), Hunting Dogs, and Hunters of Brazil. PLoS ONE.

[40] Solorio M.R., Gennari S.M., Soares H.S., Dubey J.P., Hartley A.C.Z., Ferreira F. (2010). Toxoplasma Gondii Antibodies in Wild White-Lipped Peccary (Tayassu Pecari) from Peru. J. Parasitol..

[41] Pena H.F.J., Marvulo M.F.V., Horta M.C., Silva M.A., Silva J.C.R., Siqueira D.B., Lima P.-A.C.P., Vitaliano S.N., Gennari S.M. (2011). Isolation and Genetic Characterisation of Toxoplasma Gondii from a Red-Handed Howler Monkey (Alouatta Belzebul), a Jaguarundi (Puma Yagouaroundi), and a Black-Eared Opossum (Didelphis Aurita) from Brazil. Vet. Parasitol..

[42] Dubey J.P. (2021). Outbreaks of Clinical Toxoplasmosis in Humans: Five Decades of Personal Experience, Perspectives and Lessons Learned. Parasit. Vectors.

[43] Stelzer S., Basso W., Benavides Silván J., Ortega-Mora L.M., Maksimov P., Gethmann J., Conraths F.J., Schares G. (2019). Toxoplasma Gondii Infection and Toxoplasmosis in Farm Animals: Risk Factors and Economic Impact. Food Waterborne Parasitol..

[44] Khan K., Khan W. (2018). Congenital Toxoplasmosis: An Overview of the Neurological and Ocular Manifestations. Parasitol. Int..

[45] Abdul Ameer Jaber K., Aamer Noori R. (2021). Comparisons of Toxoplasma Gondii Prevalence in Rural and Urban Areas of Al-Najaf Province of Iraq Using Serological Methods. Arch. Razi Inst..

[46] Magalhães F.J.R., Ribeiro-Andrade M., Souza F.M., Lima Filho C.D.F., Biondo A.W., Vidotto O., Navarro I.T., Mota R.A. (2017). Seroprevalence and Spatial Distribution of Toxoplasma Gondii Infection in Cats, Dogs, Pigs and Equines of the Fernando de Noronha Island, Brazil. Parasitol. Int..

[47] Rodrigues J.Y., de Almeida A.d.B.P.F., da Cruz Boa Sorte E., Gasparetto N.D., da Cruz F.A.C.S., Sousa V.R.F. (2016). Seroprevalence of Toxoplasma Gondii in Dogs of Riverside Communities of Mato Grosso Pantanal, Brazil. Rev. Bras. Parasitol. Vet..

[48] Constantino C., Pellizzaro M., de Paula E.F.E., Vieira T.S.W.J., Brandão A.P.D., Ferreira F., da Costa Vieira R.F., Langoni H., Biondo A.W. (2016). Serosurvey for Leishmania Spp., Toxoplasma Gondii, Trypanosoma Cruzi and Neospora Caninum in Neighborhood Dogs in Curitiba-Paraná, Brazil. Rev. Bras. Parasitol. Vet..

[49] Freitas A.R., Delai R.R., Kmetiuk L.B., da Silva E.C., Martini R., Brandão A.P.D., Giuffrida R., de Barros-Filho I.R., Costa da Silva R., Langoni H. (2022). Seropositivity of Anti-Toxoplasma Gondii Antibodies in Owners and Their Dogs Living on Island and Mainland Seashore Areas of Southern Brazil. Trop. Med. Infect. Dis..

[50] Mortari A.P.G., Tagarra L.G., de Souza M.L., Roman I.J., Ratzlaff F.R., Braunig P., de Andrade C.M., Cargnelutti J.F., Sangioni L.A., Vogel F.S.F. (2023). Increased Seroprevalence of Anti-Toxoplasma Gondii Antibodies in Dogs in Southern Brazil after an Outbreak of Human Toxoplasmosis. Parasitol. Res..

[51] de Macedo L.O., Bezerra-Santos M.A., Filho C.R.C.U., da Silva Sales K.G., de Sousa-Paula L.C., da Silva L.G., Dantas-Torres F., do Nascimento Ramos R.A., Otranto D. (2022). Vector-Borne Pathogens of Zoonotic Concern in Dogs from a Quilombola Community in Northeastern Brazil. Parasitol. Res..

[52] Cano-Terriza D., Puig-Ribas M., Jiménez-Ruiz S., Cabezón Ó., Almería S., Galán-Relaño Á., Dubey J.P., García-Bocanegra I. (2016). Risk Factors of Toxoplasma Gondii Infection in Hunting, Pet and Watchdogs from Southern Spain and Northern Africa. Parasitol. Int..

